# Connectivity between supplementary motor complex and primary motor cortex: a dual-coil paired-pulse TMS study

**DOI:** 10.3389/fneur.2025.1628204

**Published:** 2025-09-24

**Authors:** Hakjoo Kim, Yuming Lei, Shancheng Bao, Angelina T. Huynh, John J. Buchanan, Jessica A. Bernard, Joshua C. Brown, David L. Wright

**Affiliations:** ^1^Motor Neuroscience Lab, Division of Kinesiology, Texas A&M University, College Station, TX, United States; ^2^Brain Stimulation Mechanisms Lab, Division of Depression and Anxiety Disorders, McLean Hospital, Belmont, MA, United States; ^3^Department of Psychiatry, Harvard Medical School, Boston, MA, United States; ^4^Lifespan Cognitive and Motor Neuroimaging Lab, Department of Psychological and Brain Sciences, Texas A&M University, College Station, TX, United States

**Keywords:** transcranial magnetic stimulation, paired-pulse transcranial magnetic stimulation (ppTMS), supplementary motor complex (SMC), pre-supplementary motor area (pre-SMA), supplementary motor area (SMA), SMC-M1 circuitry, SMA-M1 paired stimulation, plasticity

## Abstract

Dual-coil paired-pulse transcranial magnetic stimulation (ppTMS) has garnered interest for its potential in elucidating neural circuit dynamics. In this study, the dual-coil ppTMS was utilized to assess the effective connectivity between the supplementary motor complex (SMC) and the primary motor cortex (M1) in humans. A robust facilitatory connection between the SMC and M1 was observed, manifested as a 19% increase in mean peak-to-peak motor-evoked potentials following conditioning of SMC 7 ms prior to M1 stimulation. Importantly, the facilitatory influence of SMC was found in subjects who received conditioning stimulation 4 cm anterior to Cz, but not in those stimulated at 5 cm, 6 cm, or 7 cm. While previous work has focused on demonstrating important temporal dynamics for SMC-M1 plasticity, the present findings highlight a critical contribution of spatial specificity for the modulation of SMC-M1 circuitry.

## 1 Introduction

The supplementary motor complex (SMC), which includes the pre-supplementary motor area (pre-SMA) and the supplementary motor area (SMA) proper, is part of the superior frontal gyrus, specifically located on the medial wall of the brain, labeled Brodmann’s area 6. For humans, this area lies anterior to the leg representation of the primary motor cortex (M1) (1, 2). Functionally, SMC has been implicated in the performance of sequential movements (2, 3). In the case of primates, findings from studies using single-unit recordings (4, 5) and through the administration of pharmacological agents (6) have highlighted the selective involvement of cells within SMC when planning and initiating motor sequences. In humans, early imaging work by Roland et al. (7) revealed heightened regional cerebral blood flow at SMC prior to executing a sequence of ballistic finger movements [see also (8, 9)]. A central role of SMC in preparing action sequences has been verified using transcranial magnetic stimulation (TMS). For example, Gerloff et al. (10) revealed disrupted performance of complex finger sequences by administering repetitive TMS (rTMS) at 15–25 Hz to SMC but not to other motor and parietal sites [also see (11), using 1-Hz rTMS; (12), using 1-Hz rTMS and continuous theta-burst stimulation; (13), using 10-Hz rTMS]. During the early phase of skill learning that involves novel sequential content, the more rostral portion of the SMC, referred to as the pre-SMA, has been identified as particularly critical, whereas the SMA proper, the more caudal segment of this region, appears more crucial for the execution of well-learned actions (14–16).

Early understanding of SMC involvement in both the control and learning of complex sequential behaviors benefited from the extensive development in neuroimaging techniques during the last few decades (17). One consequence of such advancements is the identification of intra- and inter-regional connectivity patterns that are associated with enhanced acquisition and retention of complex sequential actions. Unraveling causal relationships, however, has relied on the use of non-invasive brain stimulation tools such as TMS to uncover the direct and indirect influence of one neural site (e.g., SMC) on another (i.e., M1), referred to as effective connectivity (18, 19).

During the last 20 years, dual-coil paired-pulse TMS (ppTMS) has been used with humans to determine if a facilitatory or inhibitory tone is exerted by one region on another. For example, ppTMS has been used quite extensively to probe the influence of the cerebellum on M1, or, in other words, cerebellar-M1 connectivity (20). Early work by Ugawa et al. (21) demonstrated that administering a single TMS pulse at the cerebellum 5 ms before a second separate pulse was delivered at M1 led to a down-regulation in the excitability at M1, manifested as a reduction in the amplitude of motor-evoked potential (MEP) when compared to a condition that involved stimulation of M1 only. Since these initial efforts, ppTMS has been employed to detail a host of inter- and intra-hemispheric interactions involving M1 and a variety of neural regions that make significant contributions to the performance of action sequences [see (20), for a detailed review of this work].

Despite the general acceptance that SMC-M1 connectivity has functional relevance, there are only a modest number of studies that have adopted ppTMS to probe this part of the cortico-motor network. To date, ppTMS studies that were designed to examine the influence of SMC on M1 have mostly focused on detailing the temporal dynamics of this circuitry. For example, Arai et al. (22) offered preliminary evidence for timing-dependent plasticity of the SMC-M1 cortical network that persisted for up to 30 min. Specifically, they demonstrated increased MEP amplitude when suprathreshold stimulation of SMC preceded M1 stimulation by 6 ms but not 15 ms. Subsequent work extended the findings of Arai and colleagues, revealing that SMC’s facilitatory influence on M1 at an inter-stimulus interval (ISI) of 6 ms was more pronounced for younger as opposed to older adults. Recently, Rurak et al. (23) revealed that an ISI of 7 ms resulted in the most reliable facilitatory impact of SMC on M1 for both young and older individuals. Importantly, the magnitude of facilitation impacted by SMC on M1 appears to be functionally relevant as it was positively correlated with bimanual performance (24).

In the aforementioned studies, the examination of the temporal dynamics of SMC-M1 connectivity generally adopted a distance of ~4 cm anterior to Cz in the international 10–20 system as the appropriate anatomical location of SMC for the administration of exogenous stimulation on the surface of the skull used as a part of the ppTMS protocol (22, 25, 26). In two studies (22, 24), the conditioning stimulation at SMC during ppTMS was administered at ~7 cm anterior to Cz to probe potential pre-SMA modulation of M1. In both cases, the anticipated change in the MEP amplitude when compared to M1 stimulation alone was absent, suggesting topographic specificity for the facilitatory effect induced by SMC conditioning. The present work extended the investigation of the spatial specificity of SMC modulatory influence on M1. Specifically, the distance from Cz at which the conditioning stimulation was administered prior to M1 stimulation was systematically manipulated from 4 cm to 7 cm anterior to Cz in 1-cm steps. For all conditions, an ISI of 7 ms was used between the conditioning and test stimuli [see (23)]. First, congruent with previous findings (23–25, 27, 28), it was expected that the MEP amplitude following conditioning at 4 cm would be increased, verifying the facilitatory tone SMC exerts on M1. Second, it was anticipated that any modulation of M1 excitability via conditioning of SMC when administered at 5, 6, or 7 cm would exert a smaller impact on the resultant MEP amplitude observed at M1 [see (22, 24)].

## 2 Materials and methods

### 2.1 Participants

63 right-handed undergraduate students (45 females and 18 males, mean age ± SD: 19.78 ± 1.22, age range: 18 to 22) from the Department of Kinesiology and Sport Management at Texas A&M University participated in this study. Prior to participating in this study, all participants provided written informed consent, which was approved by the Texas A&M University Institutional Review Board. Each individual also completed the short version (29) of the Edinburgh Handedness Inventory (30) and a prescreening form for TMS. None of the individuals had metallic hardware on their scalp, cardiac pacemakers, implanted medication pumps, intracardiac lines, or central venous catheters. Additionally, they had no history of cortical stroke, other cortical lesions such as brain tumors, seizures, epilepsy, or previous brain surgeries. None had electrical, mechanical, or magnetic implants, nor did they suffer from uncontrolled migraines. Participants were not on prescription medications for brain-related disorders, did not have unstable medical conditions, and had no metal on their body or clothing above the shoulders. None of the individuals were professional musicians. This study was performed in accordance with the Declaration of Helsinki, and no adverse effects of single-pulse or ppTMS protocol were reported either during or after their participation. Upon completion of the study, participants received course credit for an undergraduate kinesiology class. Table 1 shows the number of participants in each group (4 cm: *n* = 21; 5 cm: *n* = 20; 6 cm: *n* = 15; 7 cm: *n* = 7) and the demographic information of the current study.

**Table 1:** Demographic information.

Characteristics	4 cm	5 cm	6 cm	7 cm	Total	Test statistic, *p*
*n* (Total)	21	20	15	7	63	*χ*^2^(3) = 4.865, *p* = 0.182
*n* (Female)	14	13	14	4	45	
*n* (Male)	7	7	1	3	18	
Age (SD)	19.48 (1.12)	19.85 (1.23)	19.87 (1.30)	20.29 (1.38)	19.78 (1.22)	*χ*^2^(3) = 2.589, *p* = 0.459
N-I Distance (SD)	34.38 cm (1.17 cm)	34.98 cm (1.89 cm)	34.80 cm (1.42 cm)	36.14 cm (3.08 cm)	34.87 cm (1.78 cm)	*χ*^2^(3) = 3.045, *p* = 0.385
LPA-RPA Distance (SD)	35.64 cm (1.35 cm)	36.15 cm (1.67 cm)	35.27 cm (1.05 cm)	36.71 cm (1.70 cm)	35.83 cm (1.48 cm)	*χ*^2^(3) = 4.708, *p* = 0.194
rMT (SD)	50.48% (7.05%)	51.55% (8.43%)	53.73% (6.73%)	58.43% (5.74%)	52.48% (7.56%)	*χ*^2^(3) = 6.897, *p* = 0.075
LQ^*^ (SD)	96.43 (7.01)	95.00 (11.75)	96.67 (9.99)	98.21 (4.72)	96.23 (9.16)	*χ*^2^(3) = 0.640, *p* = 0.887

Group differences in sex ratio (categorical variable) were analyzed using the Chi-square test, while differences in continuous variables were tested using the Kruskal–Wallis test, as not all groups satisfied the assumption of normality. SD, standard deviation; N-I, nasion to inion distance; LPA-RPA, left preauricular to right preauricular; rMT, resting motor threshold (% of maximum stimulator output); LQ, laterality quotient score. ^*^−100 to −61, left handers; −60 to 60, mixed handers; 61 to 100, right handers.

### 2.2 Dual-coil paired-pulse transcranial magnetic stimulation

In this study, TMS was administered using DuoMAG MP-Dual (DEYMED Diagnostic s.r.o., Czech Republic) with a butterfly T-shaped coil (2 × 30 mm-diameter windings, 30BFT-shaped, DEYMED Diagnostic s.r.o., Czech Republic). The hotspot for the right first dorsal interosseous (FDI) muscle was determined based on the peak-to-peak MEP amplitudes obtained from pre-set-grid-based hotspot-hunting [grid spacing: 2 by 2 mm, (31)], rather than from randomly selected regions around M1. For example, after measuring the MEP amplitude at one target within the grid, the surrounding area was explored, and the search proceeded in the direction where the MEP amplitude increased. The hotspot hunting was performed with a TMS coil orientation of a 45-degree angle. After hotspot hunting, the resting motor threshold (rMT) of the FDI muscle was defined as the minimum TMS intensity to evoke a peak-to-peak amplitude of 0.05 mV in at least 5 out of 10 trials (32). Brainsight TMS navigation system (Version 2.4.3, Rogue Research, Canada) with the MNI ICBM 152 average brain was used to navigate TMS, allowing for consistent stimulation of identical target points. Using the neuronavigation system, when single-pulse TMS and ppTMS were administered at each target, both angular and twist errors were kept to less than 0.05 degrees.

**Figure 1:**
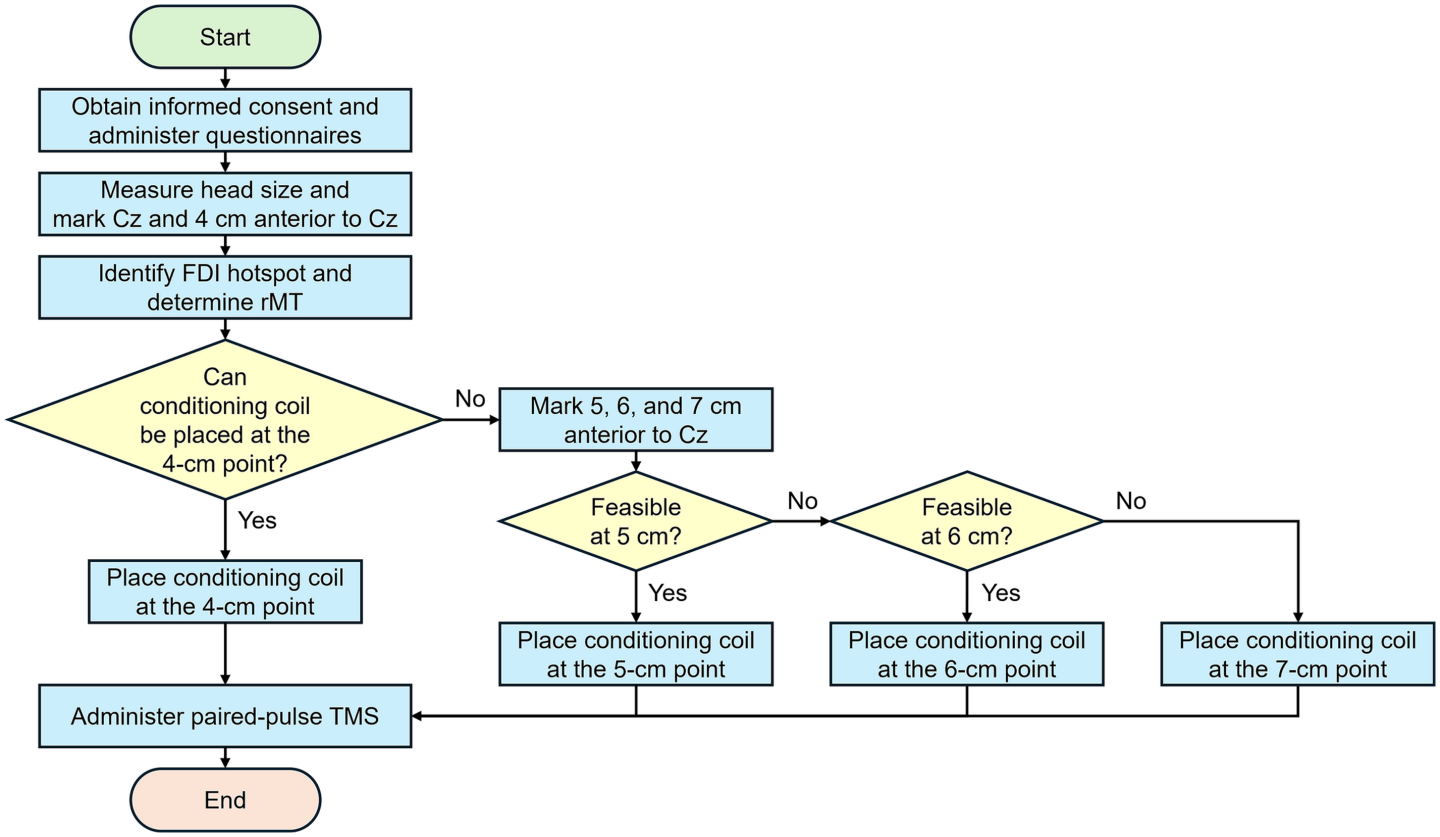
Flowchart illustrating the experimental procedure. After obtaining informed consent and administering questionnaires, an experimenter measured participants’ head size and marked Cz and a point 4 cm anterior to Cz. The right FDI muscle hotspot was identified, and the rMT was determined. If two TMS coils could not be positioned at the hotspot and the 4-cm point, additional points at 5, 6, and 7 cm anterior to Cz were also marked. After the conditioning coil location was determined, participants received paired-pulse TMS over SMC and M1. FDI, first dorsal interosseous; M1, the primary motor cortex; rMT, resting motor threshold; SMC, the supplementary motor complex; TMS, transcranial magnetic stimulation.

**Figure 2:**
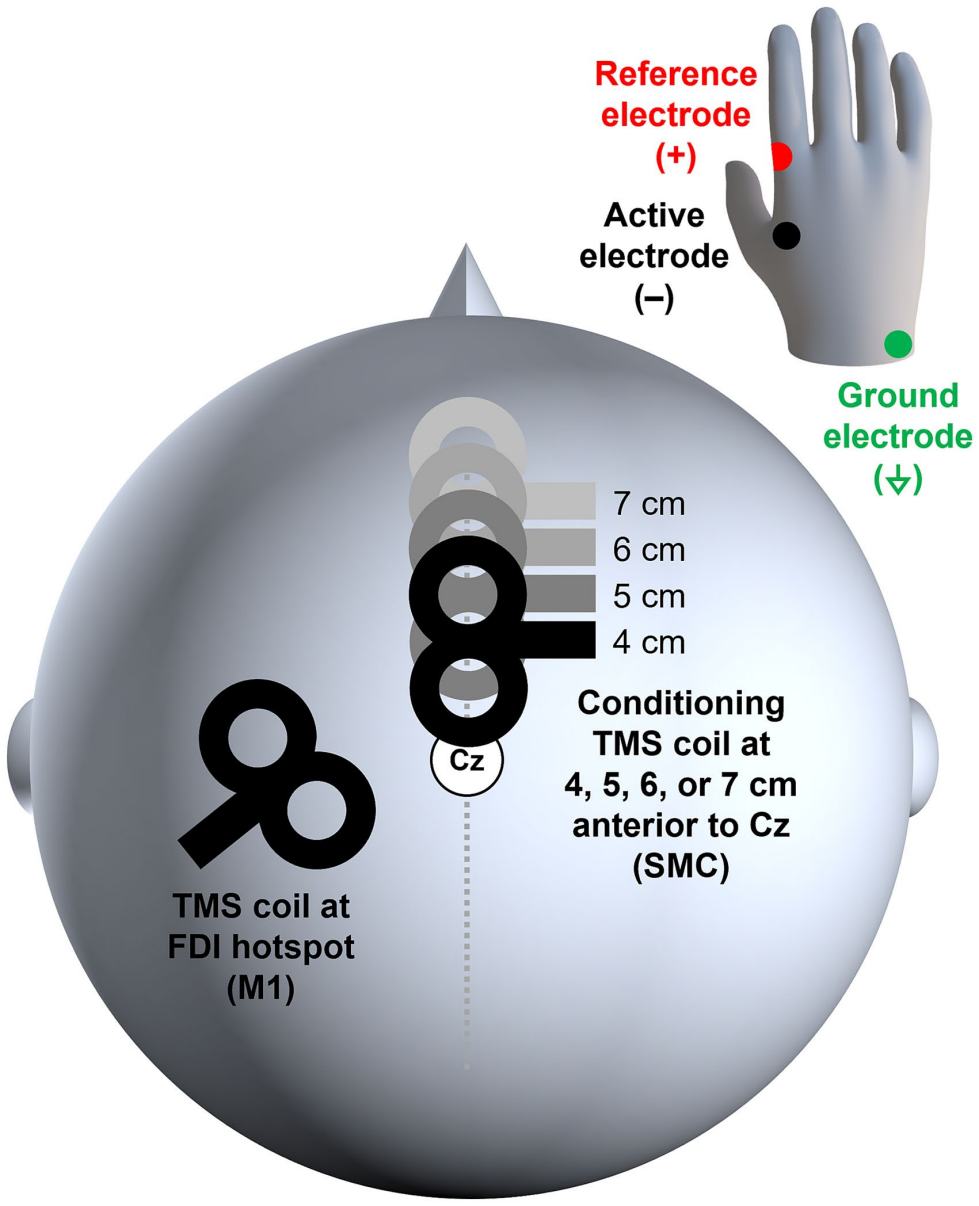
Location of the TMS coils and EMG electrodes. TMS coil for M1 stimulation was placed at the hotspot of the right FDI muscle, and the SMC conditioning coil was placed at 4, 5, 6, or 7 cm anterior to Cz. The active and reference EMG electrodes were attached to the right hand, and the ground electrode was attached over the head of the ulna on the right arm. EMG, electromyography; FDI, first dorsal interosseous; M1, the primary motor cortex; SMC, the supplementary motor complex; TMS, transcranial magnetic stimulation.

Based on the previous studies (23–25, 27, 28), SMC stimulation was administered at 4 cm anterior to Cz in the international 10–20 system. Additionally, if the hotspot of the FDI muscle was too close to an area 4 cm anterior to Cz, participants received SMC stimulation at 5, 6, or 7 cm anterior to Cz instead of 4 cm (i.e., between-subjects design) (see Figures 1, 2). The SMC stimulation (i.e., conditioning stimulus) occurred 7 ms prior to M1 stimulation (i.e., test stimulus) at an FDI hotspot (23). Participants received 30 randomly presented unconditioned (i.e., 15 single-pulse TMS at M1, unconditioned stimulus, US) and conditioned (i.e., 15 ppTMS at SMC and M1, conditioned stimulus, CS) stimuli [(27); cf. (33)] that were induced by Signal software (Version 7.04, CED Ltd., United Kingdom). The TMS intensity at the left M1 was set to 110% of the rMT [(27, 31); cf. (34)]. For the SMC, the TMS intensity was set at 140% of the rMT [(27); cf. (22–25)]. The coil was positioned at a 45°angle to the midline of the brain for M1 stimulation (27, 31), while for SMC stimulation, the coil was oriented at 270° (23, 25, 27) (see Figure 2). Participants were seated comfortably at rest during the administration of ppTMS. The mean peak-to-peak MEPs of the right FDI muscle were compared to quantify the excitability of the corticospinal pathway for both US and CS conditions.

### 2.3 Electromyography (EMG)

EMG signals from the right FDI muscle were recorded through disposable Ag-AgCl electrodes (Ambu Neuroline 720 surface electrodes, Ambu A/S, Denmark), amplified by NL844 AC Pre-amplifier (Gain × 100, Digitimer Ltd., United Kingdom), which was connected to NL820A Isolation Amplifier (Gain × 1, Digitimer Ltd., United Kingdom), filtered by NL136 Four Channel Low-Pass Filters (2 kHz, Digitimer Ltd., United Kingdom), and sampled at 5 kHz by Signal software (Version 7.04, Cambridge Electronic Design Ltd., United Kingdom). The active electrode (−) was placed over the belly of the right FDI muscle, and the reference electrode (+) was placed on the lateral aspect of the metacarpophalangeal joint of the right index finger (Figure 2). The ground electrode was positioned around the head of the ulna on the right forearm (Figure 2). The background noise was monitored in real time to ensure that it remained below 0.02 mV.

### 2.4 Procedure

Prior to participation, all individuals had submitted a signed consent form and prerequisite questionnaires. An experimenter measured each participant’s head size with a measuring tape and then located and marked two specific points on the scalp: Cz and a point 4 cm anterior to Cz (Figure 1). If the conditioning TMS coil could not be positioned at the SMC stimulation point (i.e., 4 cm anterior to Cz) due to small head size or the hotspot being too near the conditioning point, alternative spatial locations at 5, 6, and 7 cm anterior to Cz were marked (Figure 1). Participants who could not receive stimulation at the 4-cm point were then given SMC stimulation at the closest possible location to Cz among the 5, 6, or 7-cm points (Figure 1). The conditioning points (i.e., 4, 5, 6, or 7 cm anterior to Cz) were registered in the neuronavigation system by administering a minimum of three single TMS pulses. The final conditioning target was determined by averaging the locations of these pulses (i.e., the center of gravity). Single-pulse TMS was used to find a hotspot of the right FDI muscle and determine the rMT. Subsequently, participants received paired-pulse TMS at the conditioning point and the hotspot. The experimental procedure was completed within 1 h, including obtaining informed consent.

### 2.5 Statistical analyses

The primary dependent variable of the present study was the peak-to-peak MEPs of the right FDI muscle. The peak-to-peak MEPs were captured by Signal (Version 7.04, CED Ltd., United Kingdom). From 30 random US and CS presentations, raw MEPs from 15 US and raw MEPs from 15 CS were separated for statistical analysis. Among the 63 participants, individuals who received SMC stimulation at 5, 6, and 7 cm anterior to Cz were analyzed separately. MEP ratio (=CS MEP/US MEP) was calculated to compare the difference between distances of 4, 5, 6, and 7 cm. Before conducting parametric statistical analyses, such as paired samples *t*-tests and one-way analysis of variance (ANOVA), the Shapiro–Wilk test was performed to assess the normality of the data. In addition, Levene’s test was conducted to examine the homogeneity of variance prior to performing one-way ANOVA. *Post hoc* analyses following the one-way ANOVA were performed using the Scheffé method. To further assess whether the MEP ratios in each condition significantly differed from 1 (i.e., no change), one-sample *t*-tests were conducted separately for each group. Statistical analyses were conducted using SPSS (Version 28.0.0, IBM, Armonk, NY, United States). The alpha level was set at 0.05 for all tests, except for the one-sample *t*-tests across the four groups, where Bonferroni correction was applied (adjusted α = 0.0125).

## 3 Results

### 3.1 Administering a conditioning stimulus at 4 cm anterior to Cz increased M1 excitability

**Figure 3:**
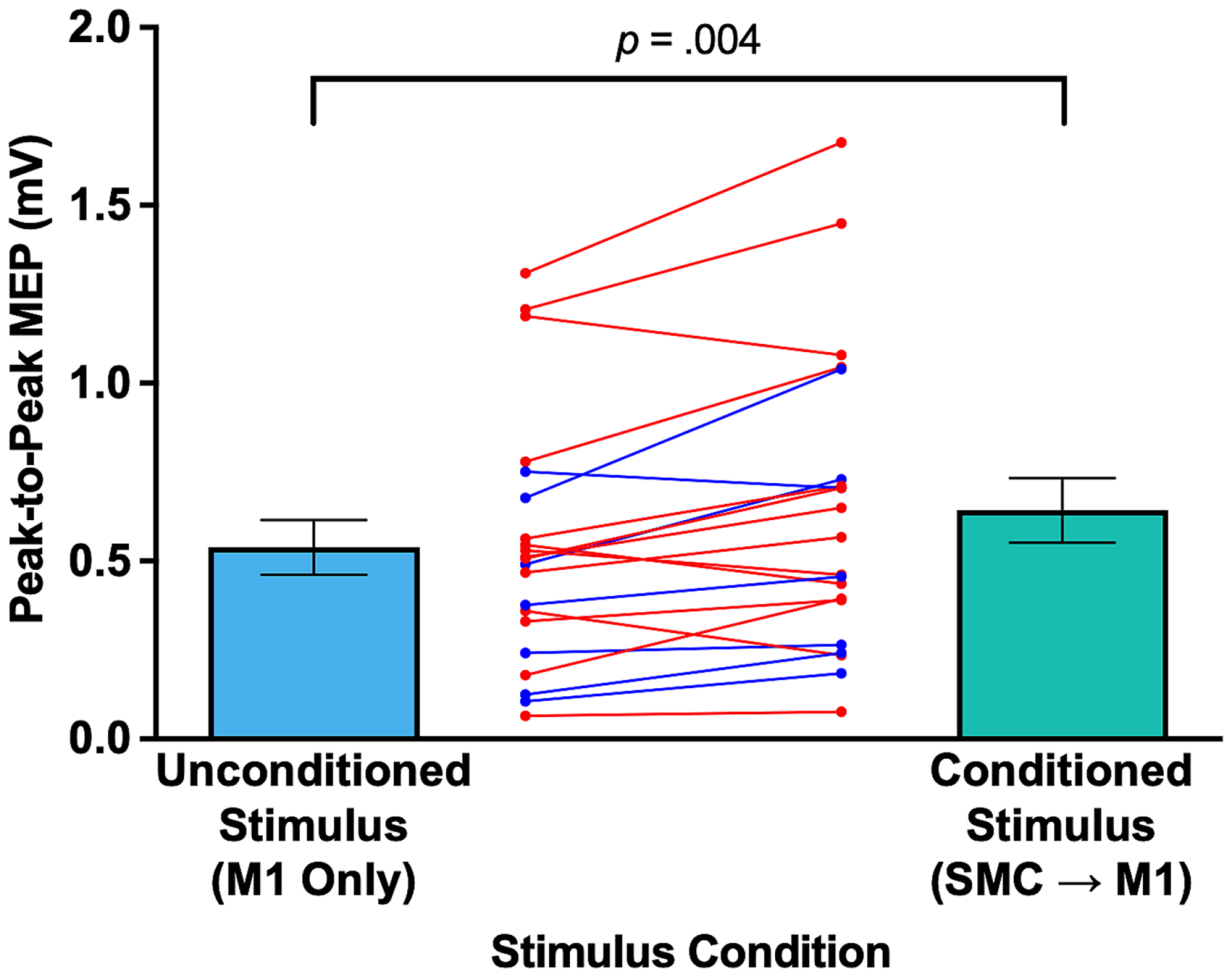
Peak-to-peak MEPs when TMS was applied at M1 only and at both SMC and M1. The left bar (blue) denotes unconditioned stimulus (i.e., single-pulse TMS at M1 only), and the right bar (mint) denotes conditioned stimulus (i.e., ppTMS at SMC prior to M1 stimulation). The red lines correspond to female participants and the blue lines to males. Error bars represent standard errors. M1, the primary motor cortex; MEP, motor-evoked potential; ppTMS, paired-pulse transcranial magnetic stimulation; SMC, the supplementary motor complex; TMS, transcranial magnetic stimulation.

The initial question addressed involved verifying that conditioning SMC at 4 cm anterior to Cz could lead to an elevation in M1 excitability. Figure 3 displays individual and mean peak-to-peak MEPs when TMS was applied at M1 only (i.e., US) and at both SMC and M1 (i.e., CS), where SMC stimulation was administered at 4 cm anterior to Cz (*n* = 21) in a manner similar to that adopted in previous studies addressing SMC-M1 connectivity (23–25). The Shapiro–Wilk test indicated that the data were normally distributed for both the US (*p* = 0.051) and CS (*p* = 0.078) conditions. The paired samples *t*-test was used to compare the mean peak-to-peak MEP amplitude for the 21 individuals who experienced the US and CS conditions at 4 cm anterior to Cz. However, this comparison was not made for the 42 individuals who received SMC stimulation at 5, 6, or 7 cm anterior to Cz. The mean peak-to-peak MEP amplitude for the US condition (mean (M) = 0.539 mV, standard error of mean (SEM) = 0.077 mV) was significantly smaller than that observed in the CS condition (M = 0.643 mV, SEM = 0.091 mV), *t*(20) = −3.229, *p* = 0.004, *d* = −0.705. The mean peak-to-peak MEP amplitude for the CS condition was 19.33% larger than for the US condition. Of the 21 individuals assessed, the peak-to-peak MEP amplitudes were increased for approximately 76% of participants when M1 was preconditioned by SMC stimulation, suggesting a robust influence of SMC on M1.

### 3.2 Administering conditioning stimulus beyond 4 cm anterior to Cz did not facilitate M1 excitability

**Figure 4:**
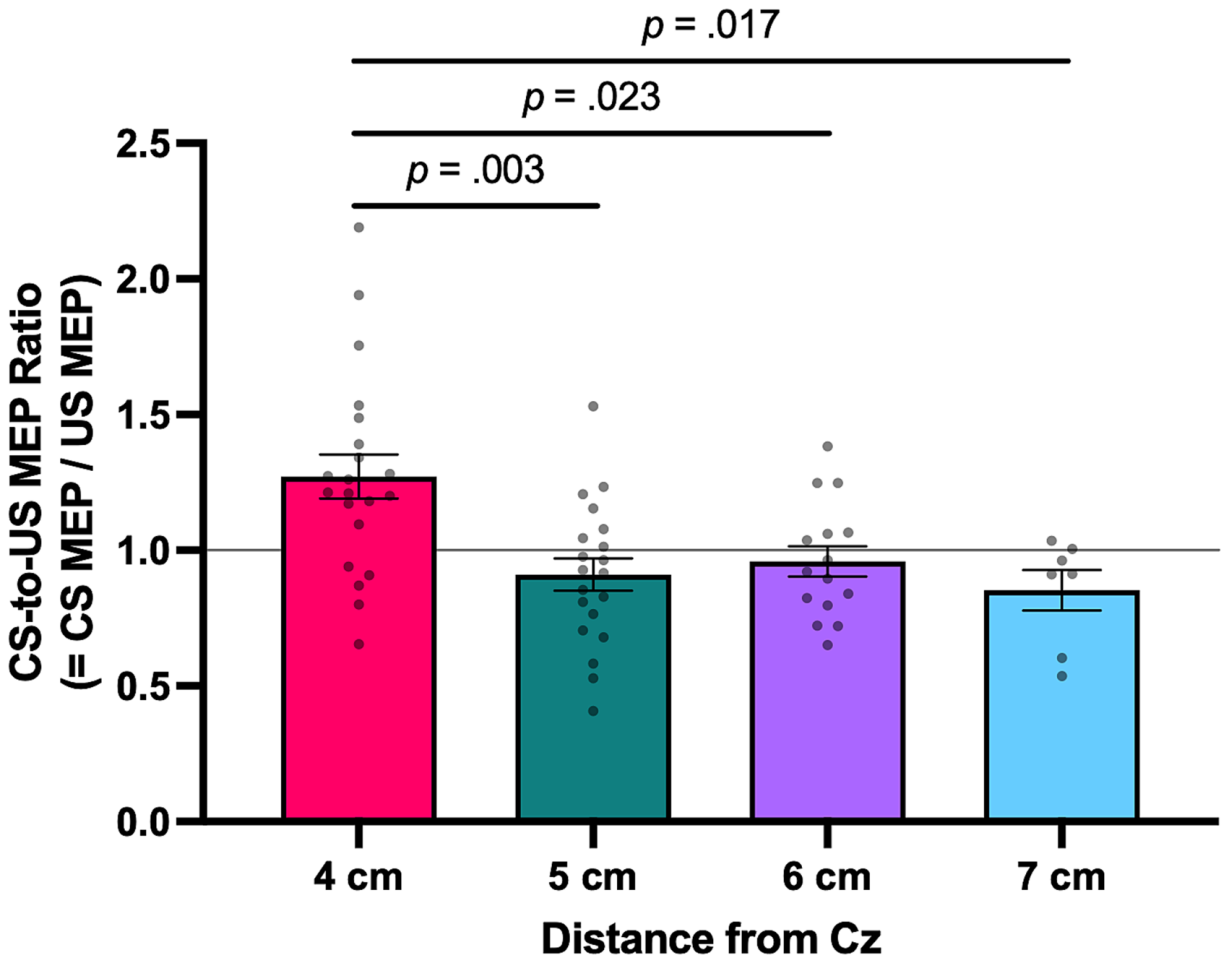
CS-to-US MEP ratios as a function of the location of conditioning stimulus relative to Cz. From left to right, the bars denote the CS-to-US ratios when SMC stimulation was administered at 4, 5, 6, or 7 cm anterior to Cz. Values greater than 1 indicate facilitation of MEP amplitude as a result of conditioning. Error bars represent standard errors. CS, conditioned stimulus; MEP, motor-evoked potential; SMC, supplementary motor complex; US, unconditioned stimulus.

A second question addressed herein was the impact of administering the conditioning stimulus in the CS condition more anterior to the standard location of 4 cm anterior to Cz used in previous work on the resultant M1 excitability. To examine this question, a CS-to-US MEP ratio was determined for each individual for each of the different locations at which the conditioning stimulus was applied in the CS condition. In this analysis, CS-to-US MEP ratios of the conditioning stimulus that was applied at 4 cm, 5 cm, 6 cm, or 7 cm anterior to Cz were analyzed. A CS-to-US ratio > 1 indicates facilitation in M1 excitability, whereas a ratio of < 1 reveals a reduction in M1 excitability as a result of conditioning. Figure 4 displays both individual and mean CS-to-US ratios as a function of the location of the conditioning stimulus relative to Cz.

It should be noted that participants were only exposed to one location for conditioning and that the 63 participants in the experiment were not evenly distributed across the conditioning locations (4 cm: *n* = 21; 5 cm: *n* = 20; 6 cm: *n* = 15; 7 cm: *n* = 7). The Shapiro–Wilk test indicated that the data were normally distributed for all conditions: 4 cm (*p* = 0.501), 5 cm (*p* = 0.369), 6 cm (*p* = 0.952), and 7 cm (*p* = 0.055). In addition, Levene’s test confirmed the homogeneity of variance across the four locations of conditioning (*p* = 0.443). The CS-to-US MEP ratio for each individual for each conditioning location was submitted to a one-way between-subject (location of conditioning) ANOVA. The one-way between factor ANOVA revealed a statistical difference in the mean CS-to-US MEP ratio as a function of the location of conditioning, *F*(3, 59) = 7.172, *p* < 0.001, partial $\eta^{2}$ = 0.267. Subsequent Scheffé *post hoc* testing revealed that the mean CS-to-US MEP ratio at the 4-cm location (M = 1.271, SEM = 0.081) was statistically larger than that observed at the 5-cm (M = 0.910, SEM = 0.059, *p* = 0.003), 6-cm (M = 0.958, SEM = 0.055, *p* = 0.023), and 7-cm locations (M = 0.852, SEM = 0.075, *p* = 0.017) (see Figure 4). The differences in CS-to-US MEP ratios between the 5, 6, and 7-cm locations did not differ significantly. Additionally, one-sample *t*-tests were conducted to determine whether the MEP ratios in each condition were statistically different from 1, which represents no change. To control for multiple comparisons across the four groups, Bonferroni correction was applied (adjusted α = 0.0125). The results indicated that the 4-cm group showed a significant increase [*t*(20) = 3.364, *p* = 0.003, *d* = 0.734], whereas the 5-cm (*p* = 0.146), 6-cm (*p* = 0.461), and 7-cm (*p* = 0.097) groups did not differ significantly from 1. These results indicate that only the group that received ppTMS 4 cm anterior to Cz exhibited a facilitatory influence on M1.

## 4 Discussion

### 4.1 Influence of SMC conditioning at 4 cm anterior to Cz on M1

An important objective of this study was to confirm the facilitatory influence of SMC conditioning at 4 cm anterior to Cz on M1, as reported in previous studies (23–25, 27, 28). Specifically, Rurak and colleagues demonstrated that administering a conditioning stimulus at 4 cm anterior to Cz, 7 ms before M1 stimulation, led to an approximate 20% increase in the peak-to-peak MEP amplitude. This facilitation of the output from M1, when preceded by SMC stimulation, has been thought to be due to the activation of glutamatergic excitatory interactions between these neural sites (6, 35, 36).

In the current study, a ppTMS protocol similar to that used by Rurak et al. (23) was employed, and as expected, the results were consistent with those previously reported (22–25, 27, 28, 37, 38). Indeed, the overall facilitatory influence of SMC conditioning on M1 was comparable to that observed by Rurak and colleagues, showing an approximately 20% increase in peak-to-peak MEP amplitude (see Figures 3, 4). It is noteworthy that the facilitatory influence of SMC on M1 observed in this study was robust, with approximately 76% of participants exhibiting this effect (see Figure 3). However, the data also suggest that the modulation of M1 activity by SMC via TMS may depend on the individual’s susceptibility to exogenous stimulation, similar to other non-invasive brain stimulation techniques, such as transcranial direct current stimulation (tDCS) (39, 40). This is reflected in the fact that a small number of individuals exhibited either no effect or actually experienced reduced M1 activity following conditioning at SMC. Such inter-individual variability may be related to differences in the microstructural properties of brain regions directly or indirectly connected to M1. For example, Kimura et al. (41) demonstrated that fractional anisotropy in both white matter tracts and gray matter areas predicted the magnitude of M1 excitability changes following intermittent theta-burst stimulation, suggesting that brain structure can influence responsiveness to stimulation. Additionally, Sydnor et al. (42) showed that the efficacy of TMS depended on the fiber density between the stimulation site and the connected region, underscoring the importance of structural connectivity. Likewise, in the present study, it is possible that individual differences in SMC–M1 structural connectivity contributed to the variability in facilitatory effects observed across participants.

An alternative explanation for our findings and those reported by Rurak et al. (23) is that the increased output from M1 in the CS condition might result from direct stimulation of M1 rather than being mediated indirectly through input from SMC, the conditioning site. This raises the possibility that the CS condition may have elicited intracortical facilitation (ICF). However, it is important to note that ICF is typically induced with an ISI of 10–15 ms (43) rather than the shorter ISI of 7 ms used in the current study and by Rurak et al. In fact, Rurak and colleagues examined the stability of the facilitatory influence of SMC on M1 across various ISIs, including 6, 7, and 8 ms, revealing that an ISI of 7 ms was the most reliable in both younger and older adults. Therefore, increasing the ISI toward the time frame frequently used to elicit ICF did not result in a change in M1 output in the study by Rurak et al.

Some additional evidence counter to a direct impact of the conditioning stimulus on M1 can be drawn from Arai et al. (22), who revealed that MEPs increased when SMC stimulation occurred 6 ms before M1 stimulation (i.e., −6 ms) while decreased when SMC stimulation was applied 15 ms after M1 (i.e., +15 ms). Perhaps more importantly, Neige et al. (38) recently reported no impact on M1 from SMC stimulation 15 ms before M1 stimulation. While it is impossible to rule out the possibility that heightened M1 excitability in the CS condition of the present experiment and others did not result from a direct influence on M1 rather than being modulated via SMC activity, the existing evidence suggests this is unlikely, given the tight temporal dynamics associated with the facilitatory effect of SMC on M1 (22, 23, 38).

### 4.2 Facilitation of M1 excitability observed only at 4 cm anterior to Cz

To date, attempts to investigate the tonic influence of SMC activation on M1 corticomotor excitability have primarily concentrated on outlining the crucial temporal dynamics of the SMC-M1 network. According to Rurak et al. (23), robust facilitation at M1 from SMC conditioning was observed with a 7-ms ISI. However, the use of a 6- or 8-ms ISI in their study resulted in significantly greater variability in corticomotor output changes in both older and younger adults.

An opportunity to examine the spatial specificity of the SMC-M1 connectivity discussed in the previous section inadvertently emerged as a result of variations in head size among participants or the specific locations of the M1 hotspots. In several cases, the standard 4-cm distance from Cz could not be used because participants’ smaller head sizes did not allow for the required distance between the two 30-mm TMS coils used to independently stimulate SMC and M1. In such cases, SMC stimulation was administered at a distance that allowed for proper placement of the two TMS coils. Consequently, a significant number of individuals used distances of 5, 6, or 7 cm anterior to Cz instead of 4 cm. This situation allowed for an evaluation of the spatial specificity of the facilitatory influence of SMC on M1 by examining the impact on M1 excitability when the conditioning stimulus was applied at these more anterior locations from Cz.

Assuming that the 4-cm site is an appropriate spatial approximation of SMC on the scalp (23–25, 27, 28) and that the SMC-M1 connectivity is responsible for the facilitatory influence discussed earlier, one would expect that applying the same conditioning stimulus at more anterior locations would result in a systematic change in corticomotor excitability observed at M1. For instance, the facilitatory influence might diminish as the conditioning site moves further away. Alternatively, it is possible that no facilitatory influence would be observed beyond the 4-cm location, indicating a high degree of spatial specificity for the impact discussed earlier and by others (22, 24). Another possibility is that the facilitatory influence of SMC conditioning on M1 might still be observed even at these more anterior sites, suggesting that M1 output is influenced by circuits extending beyond the 4-cm region anterior to Cz.

As shown in Figure 4, the CS condition involving all sites beyond 4 cm failed to increase M1 excitability. This pattern aligns with previous findings by Arai et al. (22), who compared stimulation at 4 cm and approximately 6.27 cm anterior to Cz, and Green et al. (24), who compared 4 cm and 7 cm, both reporting that facilitation was observed only at the 4-cm site. This suggests that the previously reported upregulation of M1 activity is quite focal, limited to a circuit influencing M1 from cells localized to a specific spatial location along the midline, approximately 4 cm anterior to Cz, which Rurak et al. (23) and others (24, 25, 27, 28) attribute to the SMC. Thus, it appears that cells within SMC, located at 4 cm anterior to Cz, directly influence M1, exerting a facilitatory effect that leads to a substantial increase in M1 output, as reflected in larger peak-to-peak MEPs. The upregulation of M1 excitability by SMC input seems to be highly specific in both spatial and temporal domains. While temporal specificity has been suggested in previous studies [see (22, 23)], the present study underscores the spatial specificity of SMC’s influence on M1.

### 4.3 Limitations

This study did not utilize participants’ MRIs. Therefore, it is possible that some individuals received conditioning stimulation over the pre-SMA, while others may have received it over the SMA proper. For this reason, we deliberately avoided using specific anatomical labels, such as pre-SMA or SMA proper, in the current study, and instead referred to the broader region as the SMC, which encompasses both the pre-SMA and SMA proper. To support claims regarding spatial specificity, future studies should consider using MRIs to anatomically define the conditioning target region (e.g., pre-SMA or SMA proper).

In addition, participants were not randomly assigned to groups in the current study. Instead, those who could not receive stimulation at 4 cm anterior to Cz were allocated to the 5 cm, 6 cm, or 7 cm groups. In future research, random assignment should be used to minimize potential allocation bias. Furthermore, each group should include a sufficiently large sample size, and efforts should be made to ensure better balance in sex ratios across groups.

Another important limitation is that, although approximately 76% of participants in the 4-cm group showed increased M1 excitability following SMC stimulation, this effect was not compared against a sham stimulation condition. Currently, sham stimulation over the SMC is not feasible due to the lack of small-sized TMS coils (e.g., 2 × 30 mm-diameter windings) capable of delivering effective sham pulses. However, once such coils become available, future studies should consider including a sham condition to directly evaluate the specificity and reliability of the facilitatory effect observed with real conditioning stimulation.

## Data Availability

The raw data supporting the conclusions of this article will be made available by the authors, without undue reservation.

## References

[1] Cona G Semenza C. Supplementary motor area as key structure for domain-general sequence processing: a unified account. Neurosci Biobehav Rev. (2017) 72:28–42. doi: 10.1016/j.neubiorev.2016.10.033, PMID: 27856331

[2] Nachev P Kennard C Husain M. Functional role of the supplementary and pre-supplementary motor areas. Nat Rev Neurosci. (2008) 9:856–69. doi: 10.1038/nrn2478, PMID: 18843271

[3] Tanji J. Sequential organization of multiple movements: involvement of cortical motor areas. Annu Rev Neurosci. (2001) 24:631–51. doi: 10.1146/annurev.neuro.24.1.631, PMID: 11520914

[4] Clower WT Alexander GE. Movement sequence-related activity reflecting numerical order of components in supplementary and presupplementary motor areas. J Neurophysiol. (1998) 80:1562–6. doi: 10.1152/jn.1998.80.3.1562, PMID: 9744961

[5] Shima K Tanji J. Neuronal activity in the supplementary and presupplementary motor areas for temporal organization of multiple movements. J Neurophysiol. (2000) 84:2148–60. doi: 10.1152/jn.2000.84.4.2148, PMID: 11024102

[6] Shima K Tanji J. Involvement of NMDA and non-NMDA receptors in the neuronal responses of the primary motor cortex to input from the supplementary motor area and somatosensory cortex: studies of task-performing monkeys. Jpn J Physiol. (1998) 48:275–90. doi: 10.2170/jjphysiol.48.275, PMID: 9757144

[7] Roland PE Larsen B Lassen NA Skinhoj E. Supplementary motor area and other cortical areas in organization of voluntary movements in man. J Neurophysiol. (1980) 43:118–36. doi: 10.1152/jn.1980.43.1.118, PMID: 7351547

[8] Grafton ST Hazeltine E Ivry R. Functional mapping of sequence learning in normal humans. J Cogn Neurosci. (1995) 7:497–510. doi: 10.1162/jocn.1995.7.4.497, PMID: 23961907

[9] Wiestler T Diedrichsen J. Skill learning strengthens cortical representations of motor sequences. eLife. (2013) 2:e00801. doi: 10.7554/eLife.00801, PMID: 23853714 PMC3707182

[10] Gerloff C Corwell B Chen R Hallett M Cohen LG. Stimulation over the human supplementary motor area interferes with the organization of future elements in complex motor sequences. Brain. (1997) 120:1587–602. doi: 10.1093/brain/120.9.1587, PMID: 9313642

[11] Verwey WB Lammens R van Honk J. On the role of the SMA in the discrete sequence production task: a TMS study. Neuropsychologia. (2002) 40:1268–76. doi: 10.1016/S0028-3932(01)00221-4, PMID: 11931929

[12] Verwey WB Glinski B Kuo MF Salehinejad MA Nitsche MA. Consolidation of motor sequence learning eliminates susceptibility of SMAproper to TMS: a combined rTMS and cTBS study. Exp Brain Res. (2022) 240:1743–55. doi: 10.1007/s00221-022-06358-y, PMID: 35389072 PMC8988106

[13] Kennerley SW Sakai K Rushworth MFS. Organization of action sequences and the role of the pre-SMA. J Neurophysiol. (2004) 91:978–93. doi: 10.1152/jn.00651.2003, PMID: 14573560

[14] Hikosaka O Sakai K Miyauchi S Takino R Sasaki Y Putz B. Activation of human presupplementary motor area in learning of sequential procedures: a functional MRI study. J Neurophysiol. (1996) 76:617–21. doi: 10.1152/jn.1996.76.1.617, PMID: 8836248

[15] Sakai K Hikosaka O Miyauchi S Sasaki Y Fujimaki N Pütz B. Presupplementary motor area activation during sequence learning reflects visuo-motor association. J Neurosci. (1999) 19:RC1. doi: 10.1523/JNEUROSCI.19-10-j0002.1999, PMID: 10234047 PMC6782738

[16] Sakai K Hikosaka O Miyauchi S Takino R Sasaki Y Pütz B. Transition of brain activation from frontal to parietal areas in visuomotor sequence learning. J Neurosci. (1998) 18:1827–40. doi: 10.1523/JNEUROSCI.18-05-01827.1998, PMID: 9465007 PMC6792634

[17] Kim J Lee J Hang J Kim S Lee J Kim S. Defining functional SMA and pre-SMA subregions in human MFC using resting state fMRI: functional connectivity-based parcellation method. NeuroImage. (2010) 49:2375–86. doi: 10.1016/j.neuroimage.2009.10.016 19837176 PMC2819173

[18] Derosiere G Vassiliadis P Duque J. Advanced TMS approaches to probe corticospinal excitability during action preparation. NeuroImage. (2020) 213:116746. doi: 10.1016/j.neuroimage.2020.116746, PMID: 32198049

[19] Neige C Monany DR Lebon F. Exploring cortico-cortical interactions during action preparation by means of dual-coil transcranial magnetic stimulation: a systematic review. Neurosci Biobehav Rev. (2021) 128:678–92. doi: 10.1016/j.neubiorev.2021.07.018, PMID: 34274404

[20] van Malderen S Hehl M Verstraelen S Swinnen SP Cuypers K. Dual-site TMS as a tool to probe effective interactions within the motor network: a review. Rev Neurosci. (2023) 34:129–221. doi: 10.1515/revneuro-2022-0020, PMID: 36065080

[21] Ugawa Y Uesaka Y Terao Y Hanajima R Kanazawa I. Magnetic stimulation over the cerebellum in humans. Ann Neurol. (1995) 37:703–13. doi: 10.1002/ana.410370603, PMID: 7778843

[22] Arai N Müller-Dahlhaus F Murakami T Bliem B Lu MK Ugawa Y. State-dependent and timing-dependent bidirectional associative plasticity in the human SMA-M1 network. J Neurosci. (2011) 31:15376–83. doi: 10.1523/JNEUROSCI.2271-11.2011, PMID: 22031883 PMC6703519

[23] Rurak BK Rodrigues JP Power BD Drummond PD Vallence AM. Test re-test reliability of dual-site TMS measures of SMA-M1 connectivity differs across inter-stimulus intervals in younger and older adults. Neuroscience. (2021) 472:11–24. doi: 10.1016/j.neuroscience.2021.07.023, PMID: 34333064

[24] Green PE Ridding MC Hill KD Semmler JG Drummond PD Vallence AM. Supplementary motor area—primary motor cortex facilitation in younger but not older adults. Neurobiol Aging. (2018) 64:85–91. doi: 10.1016/j.neurobiolaging.2017.12.016, PMID: 29348045

[25] Arai N Lu MK Ugawa Y Ziemann U. Effective connectivity between human supplementary motor area and primary motor cortex: a paired-coil TMS study. Exp Brain Res. (2012) 220:79–87. doi: 10.1007/s00221-012-3117-5, PMID: 22623093

[26] Lu MK Arai N Tsai CH Ziemann U. Movement related cortical potentials of cued versus self-initiated movements: double dissociated modulation by dorsal premotor cortex versus supplementary motor area rTMS. Hum Brain Mapp. (2012) 33:824–39. doi: 10.1002/hbm.21248, PMID: 21425396 PMC6870267

[27] Kim H Lei Y Huynh AT Buchanan JJ Bernard JA Brown JC. Effects of SMC iTBS on SMC-M1 connectivity and motor sequence learning. Brain Stimul. (2025) 18:1283–4. doi: 10.1016/j.brs.2025.05.059

[28] Turrini S Fiori F Bevacqua N Saracini C Lucero B Candidi M. Spike-timing-dependent plasticity induction reveals dissociable supplementary- and premotor-motor pathways to automatic imitation. Proc Natl Acad Sci. (2024) 121:e2404925121. doi: 10.1073/pnas.2404925121, PMID: 38917006 PMC11228524

[29] Veale JF. Edinburgh handedness inventory–short form: a revised version based on confirmatory factor analysis. Laterality. (2014) 19:164–77. doi: 10.1080/1357650X.2013.783045, PMID: 23659650

[30] Oldfield RC. The assessment and analysis of handedness: the Edinburgh inventory. Neuropsychologia. (1971) 9:97–113. doi: 10.1016/0028-3932(71)90067-4, PMID: 5146491

[31] Kim H Wright DL Rhee J Kim T. C3 in the 10-20 system may not be the best target for the motor hand area. Brain Res. (2023) 1807:148311. doi: 10.1016/j.brainres.2023.148311, PMID: 36889535

[32] Rossini PM Burke D Chen R Cohen LG Daskalakis Z Iorio R. Non-invasive electrical and magnetic stimulation of the brain, spinal cord, roots and peripheral nerves: basic principles and procedures for routine clinical and research application. An updated report from an IFCN committee. Clin Neurophysiol. (2015) 126:1071–107. doi: 10.1016/j.clinph.2015.02.001, PMID: 25797650 PMC6350257

[33] Cavaleri R Schabrun SM Chipchase LS. The number of stimuli required to reliably assess corticomotor excitability and primary motor cortical representations using transcranial magnetic stimulation (TMS): a systematic review and meta-analysis. Syst Rev. (2017) 6:1–11. doi: 10.1186/s13643-017-0440-8, PMID: 28264713 PMC5340029

[34] Kallioniemi E Julkunen P. Alternative stimulation intensities for mapping cortical motor area with navigated TMS. Brain Topogr. (2016) 29:395–404. doi: 10.1007/s10548-016-0470-x, PMID: 26830768

[35] Luppino G Matelli M Camarda R Rizzolatti G. Corticocortical connections of area F3 (SMA-proper) and area F6 (pre-SMA) in the macaque monkey. J Comp Neurol. (1993) 338:114–40. doi: 10.1002/cne.903380109, PMID: 7507940

[36] Muakkassa KF Strick PL. Frontal lobe inputs to primate motor cortex: evidence for four somatotopically organized ‘premotor’ areas. Brain Res. (1979) 177:176–82. doi: 10.1016/0006-8993(79)90928-4, PMID: 115545

[37] Bevacqua N Turrini S Fiori F Saracini C Lucero B Candidi M. Cortico-cortical paired associative stimulation highlights asymmetrical communication between rostral premotor cortices and primary motor cortex. Brain Stimul. (2024) 17:89–91. doi: 10.1016/j.brs.2024.01.001, PMID: 38191092

[38] Neige C Vassiliadis P Ali Zazou A Dricot L Lebon F Brees T. Connecting the dots: harnessing dual-site transcranial magnetic stimulation to quantify the causal influence of medial frontal areas on the motor cortex. Cereb Cortex. (2023) 33:11339–53. doi: 10.1093/cercor/bhad370, PMID: 37804253

[39] López-Alonso V Cheeran B Río-Rodríguez D Fernández-del-Olmo M. Inter-individual variability in response to non-invasive brain stimulation paradigms. Brain Stimul. (2014) 7:372–80. doi: 10.1016/j.brs.2014.02.004, PMID: 24630849

[40] Willmot N Leow LA Filmer HL Dux PE. Exploring the intra-individual reliability of tDCS: a registered report. Cortex. (2024) 173:61–79. doi: 10.1016/j.cortex.2023.12.015, PMID: 38382128

[41] Kimura I Oishi H Hayashi MJ Amano K. Microstructural properties of human brain revealed by fractional anisotropy can predict the after-effect of intermittent theta burst stimulation. Cereb Cortex Commun. (2022) 3:tgab065. doi: 10.1093/texcom/tgab065, PMID: 35083435 PMC8784864

[42] Sydnor VJ Cieslak M Duprat R Deluisi J Flounders MW Long H. Cortical-subcortical structural connections support transcranial magnetic stimulation engagement of the amygdala. Sci Adv. (2022) 8:eabn5803. doi: 10.1126/sciadv.abn5803, PMID: 35731882 PMC9217085

[43] Kujirai T Caramia MD Rothwell JC Day BL Thompson PD Ferbert A. Corticocortical inhibition in human motor cortex. J Physiol. (1993) 471:501–19. doi: 10.1113/jphysiol.1993.sp019912, PMID: 8120818 PMC1143973

